# Combination of Percutaneous Transhepatic Balloon-Assisted Transjugular Intrahepatic Collateral Caval Shunt and Embolization for Successful Hemostasis of Lower Gastrointestinal Bleeding Associated With Extremely Enlarged Anorectal Varicose Veins

**DOI:** 10.7759/cureus.21053

**Published:** 2022-01-09

**Authors:** Christos Sotiropoulos, Eftichia Sakka, Georgia Diamantopoulou, Georgios J Theocharis, Konstantinos C Thomopoulos

**Affiliations:** 1 Department of Gastroenterology, University General Hospital of Patra, Patra, GRC; 2 Department of Internal Medicine, General Hospital of Patra “St. Andrew”, Patra, GRC

**Keywords:** embolization, portosystemic shunt, sclerotherapy, collateral shunt, ectopic varices, variceal bleeding, portal vein thrombosis, portal hypertension

## Abstract

Ectopic varices may frequently occur in the rectum in the context of portal hypertension. Although rectal variceal bleeding is not a frequent bleeding situation, it can be life-threatening unless diagnosed and treated immediately. However, there is no specific treatment strategy established so far. We report a case of a man with extrahepatic portal hypertension and severe hematochezia due to rectal variceal bleeding. The patient was diagnosed in the past with portal vein thrombosis, in the context of myelodysplastic syndrome/myeloproliferative neoplasm overlap syndrome, with ectopic varices in the small intestine, colon, rectum and anal canal, peritoneum and perisplenic veins, treated with mesorenal shunt placement and an oral beta-blocker. After the initial stabilization with fluid replacement and red blood cell transfusion, he underwent endoscopic injection sclerotherapy, with no effect on bleeding episodes, while the large size of the varices precluded the option of endoscopic band ligation. Due to the presence of large collateral veins next to the inferior vena cava, the patient underwent combination therapy with Percutaneous Transhepatic Balloon-Assisted Transjugular Intrahepatic Collateral Caval shunt placement, to decompress portal pressure, followed by angiographic embolization of the feeding vessels resulting in successful hemostasis. Hematochezia ceased, hemoglobin was stabilized and the patient was safely discharged from the hospital. Controlling and treating rectal varices can be a challenging task indicating the need of a multidisciplinary approach. In the absence of well-established treatment guidelines for rectal varices, we highly recommend treatment of refractory ectopic variceal bleeding, non-responsive to endoscopic treatments, with portocaval shunt placement in combination with embolization.

## Introduction

Portal hypertension (PHT) may induce the formation of a collateral circulation, between portal venous system and systemic circulation, called varices [1-6]. The most common sites of varices in the gastrointestinal tract are the esophagus and the gastric fundus [1, 3, 4]. Ectopic varices are considered as dilated portosystemic collateral veins along the digestive tract other than the esophageal and gastric region [1, 3-6]. Although esophageal variceal bleeding is the most common type of varicose vein bleeding, PHT can be associated with variceal hemorrhage of other sites [2].

Anorectal varicose veins are portosystemic collaterals that develop from the communication between superior hemorrhoidal and middle or inferior hemorrhoidal veins and can be visualized via anoscopy as enlarged and tortuous submucosal veins extended from the anal canal up to the middle rectum [4, 7-9]. Their prevalence has been estimated at 38-56% in cirrhotic patients and 63-94% in those with extrahepatic portal vein obstruction [7, 10]. Bleeding of rectal varices is rare, with an incidence of 1-8%, but is the most common site of ectopic varicose veins, accounting for 44.5% of all ectopic varices [2, 4, 10]. Despite being rare, anorectal variceal hemorrhage may be life-threatening unless diagnosed and treated in time [2].

Currently, there have not been established specific diagnostic workup and treatment guidelines for rectal variceal bleeding [1]. The available treatment options include vasoactive drugs, endoscopic injection sclerotherapy (EIS), endoscopic band ligation (EBL), transjugular intrahepatic portosystemic shunt placement (TIPS), balloon-occluded retrograde transvenous obliteration (BRTO) and angiographic embolization with cyanoacrylate alone or a combination of cyanoacrylate and coils, while surgery is being considered as an option for refractory bleeding rectal varices [1, 3, 4, 6, 11, 12].

In this case, we present a patient who had portal vein thrombosis (PVT), PHT and continued to have rectal bleeding due to anorectal variceal hemorrhage, despite a patent splenorenal shunt, to highlight awareness of this unusual condition. The patient presented with lower gastrointestinal bleeding initially treated unsuccessfully with EIS followed by a combination of portosystemic shunt placement and embolization in order to achieve hemostasis.

## Case presentation

**Figure 1:**
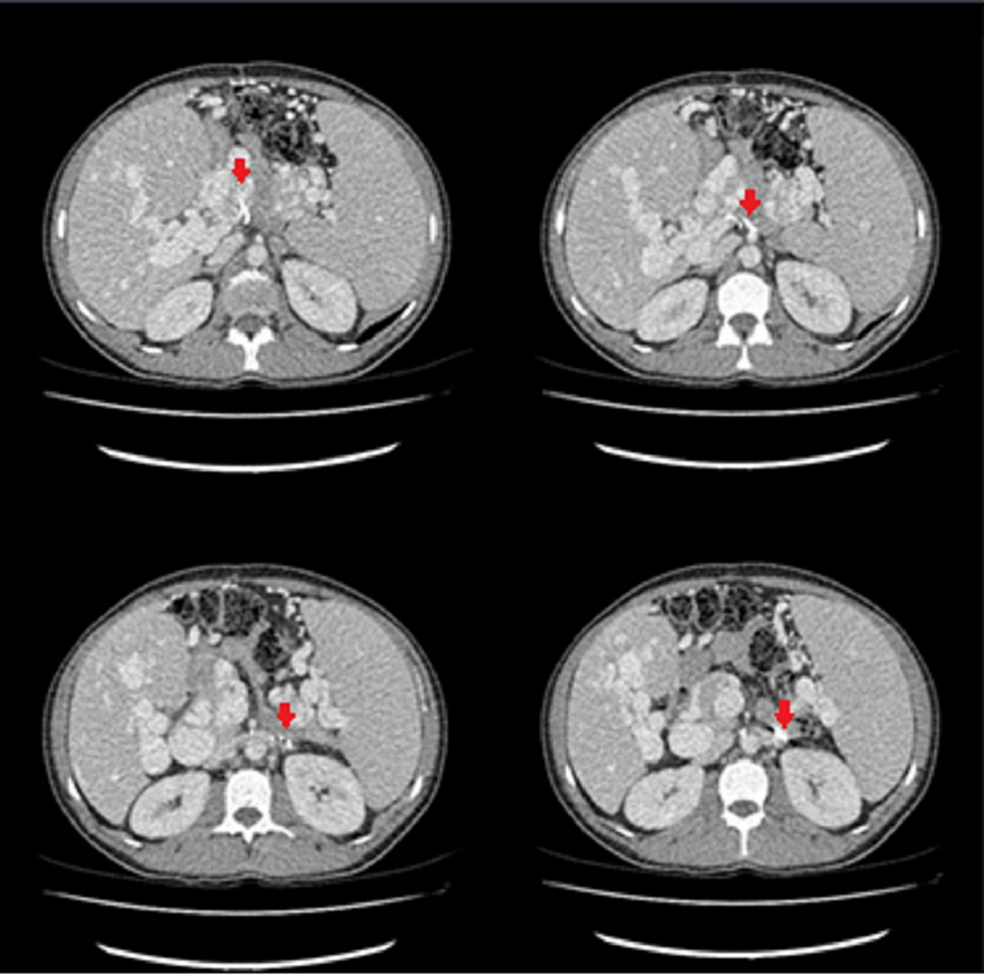
Computed tomography showing mesenteric-inferior-renal shunt placement (red arrows).

In April 2021, a 43-year-old man with known PVT and ectopic varices was admitted to our hospital due to one-week history of severe hematochezia. His past medical history included extrahepatic PHT due to PVT, in the context of myelodysplastic syndrome (MDS)/myeloproliferative neoplasm (MPN) overlap syndrome, with ectopic varices in the small intestine, colon, rectum and anal canal, peritoneum and perisplenic veins, treated with mesenteric-inferior-renal shunt placement (Figure 1, red arrows) and an oral beta-blocker (carvedilol 6.25 mg b.i.d.).

The patient was hemodynamically stable and the physical examination revealed splenomegaly and a digital rectal examination was positive for blood, without obvious hemorrhoids. Laboratory analyses demonstrated anaemia (serum hemoglobin of 7.9 g/dl), thrombopenia (blood platelets 94 x 1000/mm³), normal prothrombin time, normal kidney function (serum creatinine level 1.0 mg/dl) and normal liver enzymes, without signs of inflammation.

**Figure 2:**
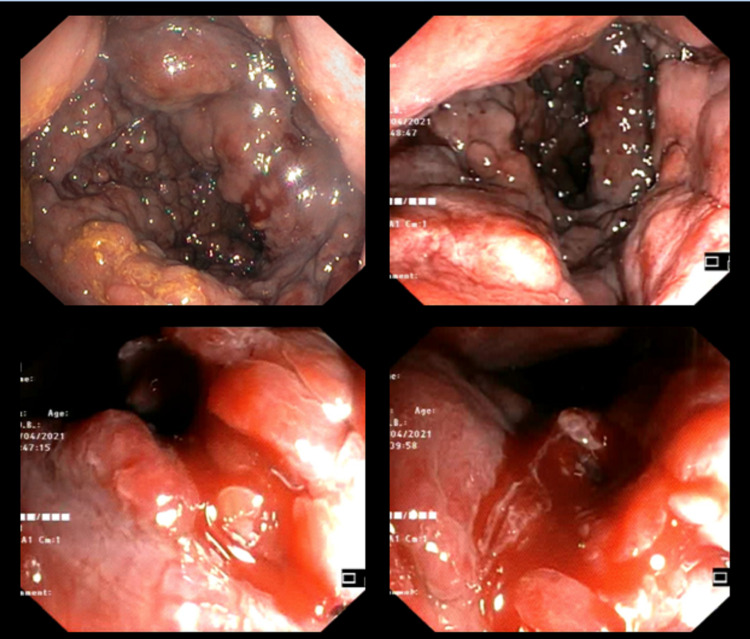
Colonoscopy revealing active lower intestinal bleeding originating from extremely enlarged anorectal varicose veins.

**Figure 3:**
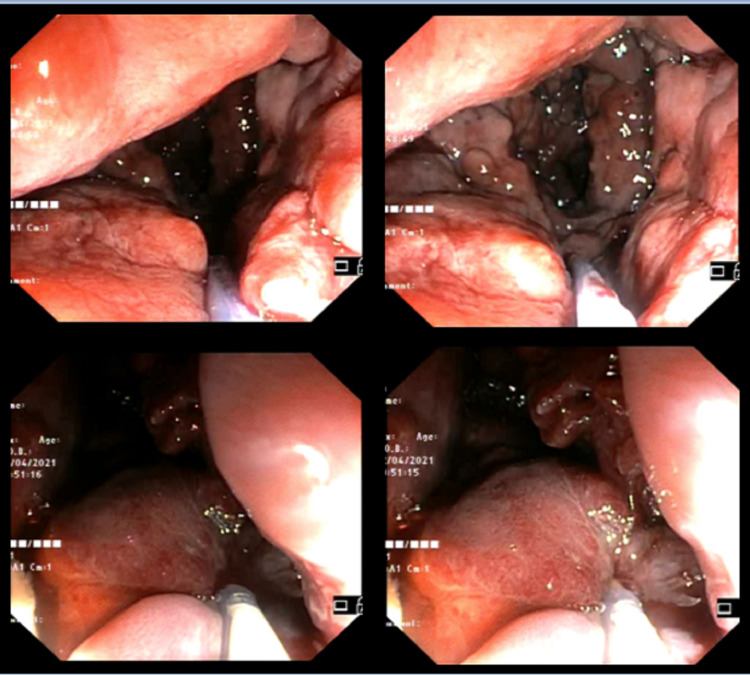
Endoscopic injection sclerotherapy with synthetic biodegradable cyanoacrylate basis glue.

Initially, the patient was conservatively treated with fluid resuscitation and blood transfusions. Additionally, an intravenous somatostatin solution was administered to reduce the portal pressure. A left colonoscopy immediately followed revealing active lower intestinal bleeding originating from extremely enlarged anorectal varicose veins (Figure 2) and EIS with synthetic biodegradable cyanoacrylate basis glue was performed (Figure 3).

**Figure 4:**
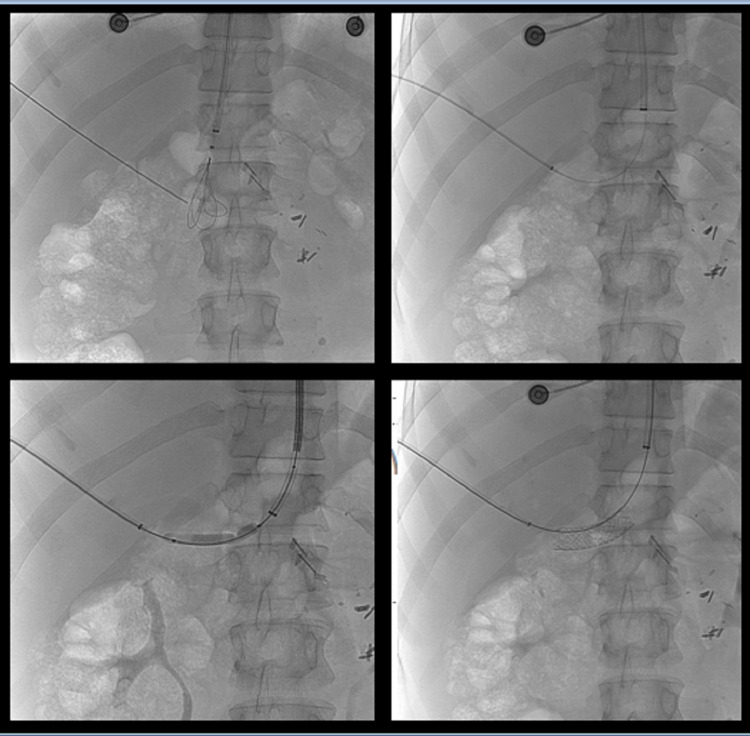
Percutaneous Transhepatic Balloon-Assisted Transjugular Intrahepatic Collateral Caval shunt placement.

Endoscopic injection sclerotherapy was unsuccessful to achieve hemostasis with the patient continuing to have bleeding episodes and EBL was not an option due to the size of the varices. Therefore, in collaboration with the department of interventional radiologists, the patient underwent portosystemic shunt placement in order to achieve portal decompression and reduce the likelihood of rebleeding. Due to the presence of large collateral veins next to the inferior caval vein, we preferred to carry out Percutaneous Transhepatic Balloon-Assisted Transjugular Intrahepatic Collateral Caval shunt placement (Figure 4) instead of the classic TIPS procedure.

**Figure 5:**
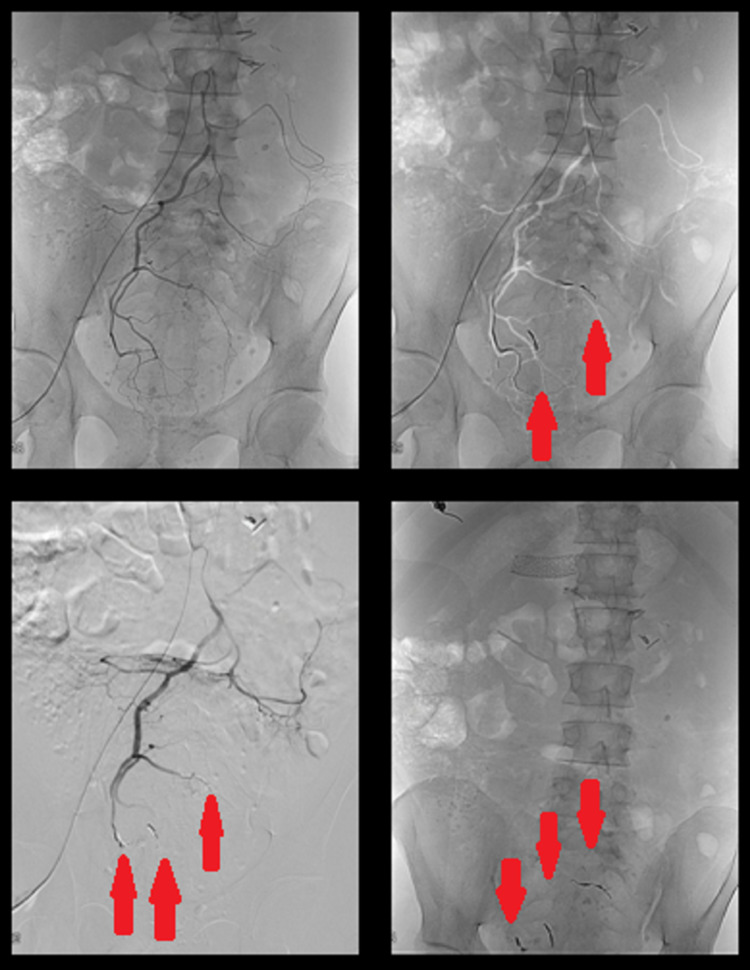
Angiographic embolization.

In the next few days, hematochezia continued, a passable stent was checked with computed tomography and the patient underwent angiographic embolization (Figure 5).

Postoperatively, the patient’s hematochezia ceased and his hemoglobin concentration significantly increased to 9.5 g/L with no need for additional blood transfusions. The patient was then discharged with instructions for continuation of the treatment with beta-blocker and repeating a colonoscopy in a few weeks.

## Discussion

The attempt to control and treat anorectal variceal hemorrhage can be a very challenging task [8, 12]. So far, there are no specific guidelines for rectal variceal rupture, thus, a multidisciplinary approach is the most appropriate strategy including conservative, endoscopic, radiological and surgical measures [1,2]. Initial management of ectopic variceal bleeding includes resuscitation and stabilization of the patient with fluid and red blood cell transfusion, urgent localization of the bleeding site or source and suitable direct therapy [1, 8, 9].

In haemodynamically stable patients, endoscopic treatment is the most preferred option as it is less invasive and readily available [12]. Interventional therapy, such as EIS with histoacryl, cyanoacrylate, ethanolamine oleate, sodium tetradecyl sulfate, polidocanol or thrombin may successfully control bleeding [1, 2]. On the other hand, EBL is an appropriate option when the diameter of the caliber of the endoscope is bigger than the varicose vein [2]. It has been recommended to limit the use of EBL to ectopic varices with a diameter less than 15 mm because of the risk of developing a wide defect in the varix by not banding the entire varix [1]. In addition, EIS has been reported to be more effective in the management of active bleeding from rectal varices with a smaller rebleeding rate [12]. In our case, we preferred EIS because of the higher efficacy and because of the large size of the anorectal varices with an increased risk of post-banding bleeding [12].

Although transjugular intrahepatic portosystemic shunt placement is the gold standard treatment of the upper gastrointestinal variceal bleeding refractory to endoscopic therapy, its therapeutic role in ectopic variceal bleeding has only been described in small case series and case reports [12]. TIPS placement achieves decompression of the portal venous system and prevention of recurrent variceal bleeding [2], and may be useful in controlling bleeding from rectal varices, but it may not always be successful in controlling massive bleeding, even after normalization of portal hypertension [12]. Therefore, in some cases as ours, a combination of endoscopic and radiologic procedures, such as portocaval shunt placement and rectal variceal embolization, is necessary [12].

Direct percutaneous access to varices is possible by interventional radiological techniques with subsequent injection of different kind of materials like thrombin, gel foam, steel coils, collagen or autologous blood clot [1]. Embolization therapy does not affect directly the bleeding problem, but will occlude the feeding vessel of the bleeding ectopic varix and so it can be an indirect treatment of ectopic variceal bleeding with success rates ranging from 80% up to 94% [1]. Because this technique does not resolve PHT as the origin of the ectopic varices neither decompresses the portal venous system, high rebleeding rates up to 65% within five months are reported, and repeating of the procedure or subsequently TIPS is often required [1].

If all other therapeutic strategies have failed, surgical management remains an option to be considered as a treatment for ectopic variceal bleeding [1]. However, it is not recommended in patients with deteriorated liver function and liver cirrhosis due to the increased operative risk from the underlying liver disease [7]. Thus, surgical management could be considered the appropriate choice in patients with an extrahepatic PVT and preserved hepatic reserve on the failure of non-surgical methods [10].

Unlike as in gastroesophageal or colonic variceal hemorrhage, no solid evidence is available for the routine use of somatostatin, octreotide or terlipressin [1, 8]. Whether the role of vasoactive agents in the initial treatment of ectopic variceal bleeding is the same as in bleeding from esophagogastric varices is currently not well established [1]. When we address to the role of using beta-blocking agents in the primary and secondary prophylaxis of ectopic variceal bleeding, again, we are faced with limited data, but their efficacy seems plausible, especially after other therapeutic management [1]. Even though, no recommendations support primary prophylaxis in order to prevent rebleeding from rectal varices empirical use of a beta-blocker is usually tried [12].

Our case highlights a challenging scenario in which rectal varices were too large for EBL, EIS was unsuccessful and collateral caval shunt placement was performed to control intractable rectal variceal bleeding. Nevertheless, shunt achieved incomplete hemostasis, so variceal embolization was performed to achieve successful hemostasis. This case is being reported as the second case of a collateral caval shunt placement in the published literature, after the first described by Hau et al. in 2014 [6].

## Conclusions

The management of a patient presenting with ectopic variceal bleeding should be individualized and multidisciplinary. Presently, there do not exist well-established therapeutic strategies and diagnostic workups for ectopic variceal hemorrhage. As there are no established guidelines to define the appropriate management strategies for rectal varices and most data nowadays available are extrapolated from small studies of case reports, further randomized controlled trials and investigation of case series are required to establish a standard treatment strategy.
